# Pneumoperitoneum in a Patient With COVID-19 and Acute Respiratory Distress Syndrome Without Pneumothorax

**DOI:** 10.7759/cureus.39593

**Published:** 2023-05-28

**Authors:** Kartik K Goswami, Rakesh Kumar, Sanjeev K Goswami

**Affiliations:** 1 College of Medicine, California Northstate University College of Medicine, Elk Grove, USA; 2 Pulmonary and Critical Care Medicine, Stockton Pulmonary Doctors, Stockton, USA; 3 Pulmonary and Critical Care Medicine, St. Joseph’s Medical Center, Stockton, USA

**Keywords:** pulmonology, covid-19 complications, rare, ventilator, sob - shortness of breath, pulmonary barotrauma, ards, pneumoperitoneum, pneumothorax (ptx), covid-19

## Abstract

We present a case report of pneumoperitoneum, pneumomediastinum, and subcutaneous emphysema in a patient with COVID-19 pneumonia-causing acute respiratory distress syndrome (ARDS) without any pneumothorax occurring. Pneumothorax, pneumomediastinum, and subcutaneous emphysema are known complications of barotrauma due to positive pressure from mechanical ventilation which is necessary for patients suffering from a severe case of COVID-19. In our literature search, we could not find any reported case of pneumoperitoneum without pneumothorax occurring. Our case is an important addition to the literature presenting a rare complication of mechanical ventilation in patients with ARDS.

## Introduction

The inspiration for this case report originated from a thought-provoking conversation in which a rare and intriguing case was brought to our attention. The significance of this case lies in its ability to shed light on an unusual complication: severe pneumoperitoneum resulting from mechanical ventilation, specifically in the context of COVID-19-induced severe damage. In the realm of positive pressure ventilation-related complications, the occurrence of pneumoperitoneum without pneumothorax is a rare phenomenon that challenges our understanding of the interplay between COVID-19, barotrauma, and its associated sequelae [1,2].

Understanding the pathophysiology and risk factors associated with pneumoperitoneum in the absence of pneumothorax can aid clinicians in managing COVID-19 patients receiving mechanical ventilation. Insights gleaned from this case can inform strategies for optimizing ventilation protocols and implementing preventive measures, potentially reducing the occurrence of rare complications such as pneumoperitoneum.

## Case presentation

**Figure 1:**
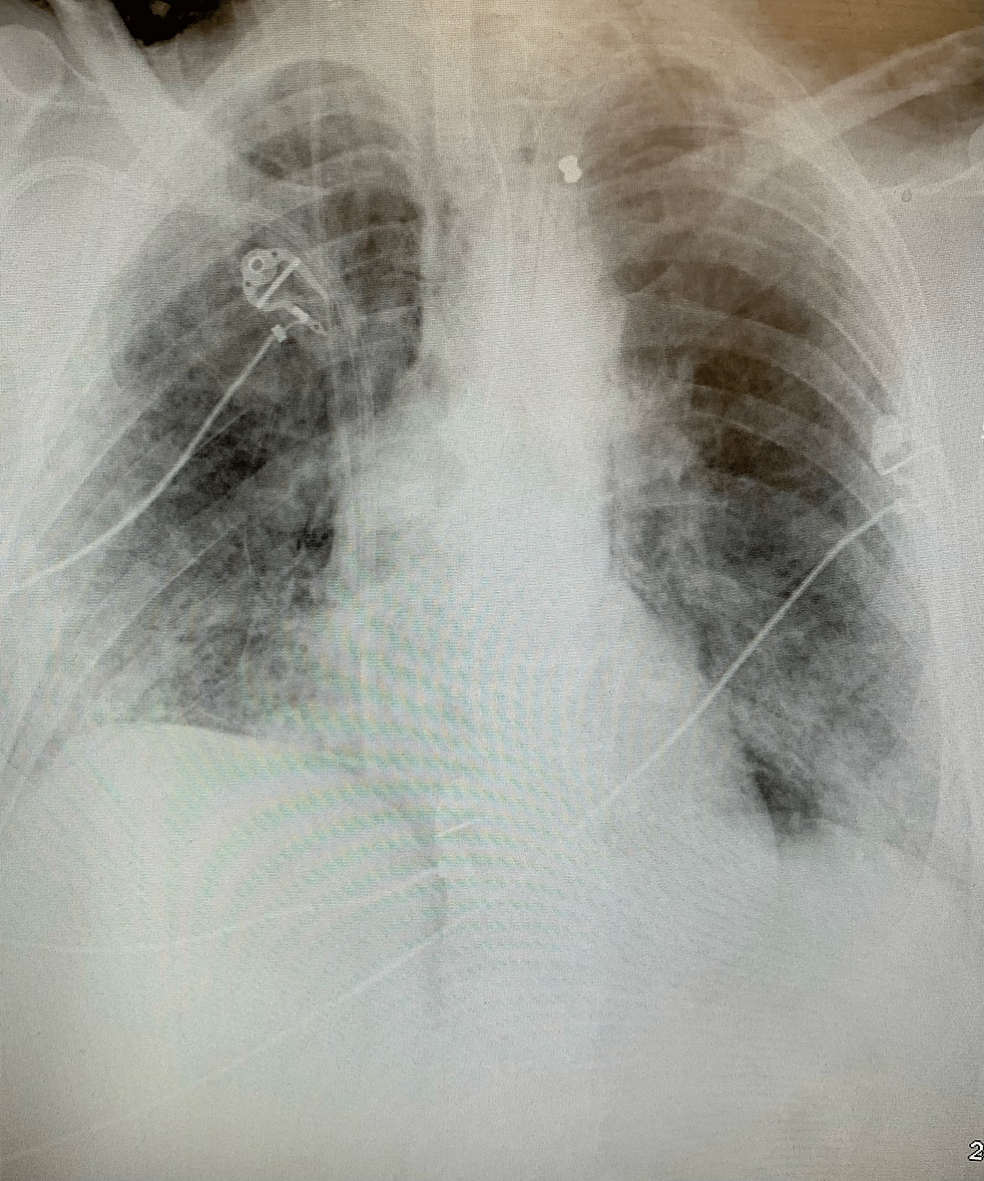
Chest X-ray showing no pneumothorax in the patient.

A 56-year-old male with a past medical history significant for hypertension and hyperlipidemia presented to the hospital with complaints of shortness of breath for four days. He tested positive for COVID-19 on a reverse transcriptase polymerase chain reaction (RT-PCR) test a few days before coming to the hospital. In the emergency room, his pulse oximetry reading was 96% on room air, temperature was 36.5 Celsius, and blood pressure was 140/89 mmHg. Initial laboratory data were significant for an elevated white blood cell count of 13000 per microliter, and chemistry was normal. He was admitted to the hospital with COVID-19 pneumonia. He was started on empiric antibiotics, dexamethasone, and remdesivir. His oxygen saturation started dropping and was having significant respiratory distress. He was put on non-invasive pressure ventilation. On the second day of his hospitalization, he developed significant respiratory distress (pH: 7.36, pO_2_: 60mmHg on 100% FiO_2_, and PCO_2_: 38 mmHg) requiring intubation and mechanical ventilation. He was ventilated with low tidal volume, 6 ml/kg predicted body weight as per current guidelines, acute respiratory distress syndrome (ARDS) net trial, and his peak pressure was in the range of 40-42. Prone therapy for 16 hours a day was started due to his low arterial pO_2_ divided by the fraction of inspired oxygen (P/F ratio of 60 mmHg). On the thirteenth day of his hospitalization, chest X-ray showed no pneumothorax as seen in Figure 1. 

**Figure 2:**
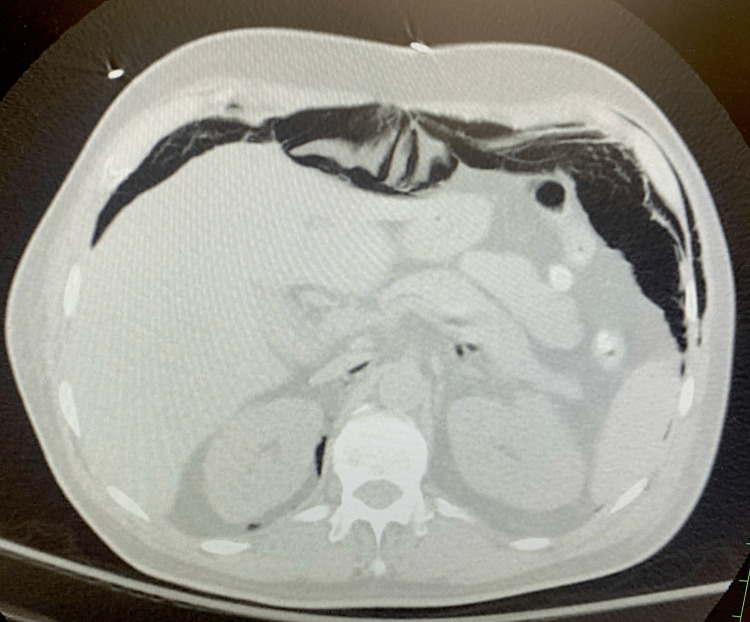
CT scan showing pneumoperitoneum in the patient.

**Figure 3:**
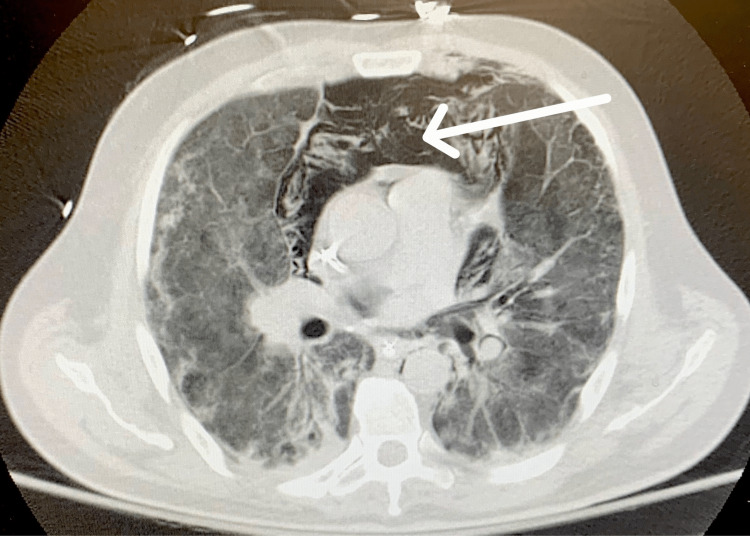
CT scan with an arrow pointing to pneumomediastinum.

This occurred without any change in peak pressure or other clinical parameters that normally occur with pneumomediastinum and pneumothorax. Chest X-ray was repeated later that day which showed no significant changes. Another chest X-ray was done the next day, and it showed free air under the diaphragm. A CT scan of the neck, chest, and abdomen was done which showed extensive subcutaneous emphysema, pneumomediastinum, and pneumoperitoneum without any pneumothorax again as seen in Figure 2. Another CT scan was done which showed pneumomediastinum occurring within the patient (Figure 3).

Consultations with general surgery and cardiothoracic surgery were obtained, and no surgical intervention was recommended for this patient. The patient was monitored with daily chest X-rays which showed no obvious pneumothorax. The patient expired after 26 days in the hospital due to complications of COVID-19 infection and multiple organ failure.

## Discussion

Pneumoperitoneum can occur in COVID-19 patients due to possible lung complications caused by the disease leading to air leakage into the peritoneal cavity [3]. This pathology is caused by the presence of air within the peritoneum cavity due to some external cause leading to bleeding, subcutaneous hemorrhage, and vascular hemorrhage in the abdomen. Unfortunately, despite the best efforts of the medical team, our patient succumbed to the complications arising from his extensive pneumoperitoneum, pneumomediastinum, and subcutaneous emphysema, despite the absence of pneumothorax.

In our comprehensive literature search, spanning various medical databases, we were unable to uncover any reported cases of pneumoperitoneum occurring in the absence of pneumothorax [4-6]. Pneumomediastinum can occur due to a combination of barotrauma and alveolar damage due to SARS-CoV-2 infection. Pneumomediastinum can develop after alveolar membrane damage and rupture, followed by air dissection through the bronchovascular sheath into the mediastinum [7]. This highlights the uniqueness and rarity of our patient's condition, as well as the limited knowledge available regarding this specific complication.

It is crucial to recognize that pulmonary barotrauma, including the aforementioned complications, can significantly impact the course of mechanical ventilation, leading to prolonged periods of ventilation support and extended stays in the intensive care unit [4,5]. These complications pose substantial challenges to patient management and necessitate close monitoring and careful consideration of the ventilatory strategies employed.

The knowledge derived from this case underscores the need for ongoing research and collaboration within the medical community. By further investigating the underlying mechanisms and risk factors contributing to pneumoperitoneum without pneumothorax, we can broaden our understanding of the complex interplay between mechanical ventilation, COVID-19, and associated complications. Additionally, future studies may focus on developing strategies to prevent or mitigate these rare complications, ultimately improving patient care and outcomes.

## Conclusions

This case report emphasizes a crucial aspect of extrapulmonary air accumulation, specifically pneumoperitoneum extending from an intrathoracic source, in mechanically ventilated patients with ARDS, particularly those affected by COVID-19 infection. Recognizing and comprehending its underlying pathophysiology would reduce the necessity for unwarranted general surgical consultations and exploratory laparotomy. This article significantly enhances the existing database and makes a valuable contribution.
